# Advances in the management of obesity: non-pharmacological strategies, herbal formulations, current pharmacotherapy, emerging drugs, and future perspectives

**DOI:** 10.3389/fendo.2026.1933913

**Published:** 2026-09-07

**Authors:** Kaushal Navanath Mehetre, Jayshree Shriram Dawane

**Affiliations:** Department of Pharmacology, Bharati Vidyapeeth (Deemed to be University) Medical College, Pune, India

**Keywords:** anti-obesity drugs, clinical trials, GLP-1 receptor agonists, obesity, pharmacotherapy, retatrutide, tirzepatide, weight management

## Abstract

Obesity is a long-term, complex disorder characterised by excessive adiposity that increases the risk of metabolic and cardiovascular diseases. Obesity is a significant health problem, with its prevalence increasing steadily over the last several decades. This rising trend has contributed substantially to the burden of metabolic, cardiovascular and other chronic diseases. Lifestyle modification remains the primary approach to obesity treatment; long-term weight reduction is difficult to achieve and sustain due to complex neurohormonal mechanisms that regulate appetite and energy homeostasis. Consequently, pharmacotherapy has become an essential component of comprehensive obesity management in appropriately selected patients. Recent advances in obesity pharmacology have revolutionised treatment strategies, particularly with the introduction of incretin-based therapies such as glucagon-like peptide-1 (GLP-1) receptor agonists and dual glucose-dependent insulinotropic polypeptide (GIP)/GLP-1 receptor agonists, which have demonstrated unprecedented efficacy in achieving clinically meaningful and sustained weight loss. Furthermore, several novel agents, including triple agonists, oral peptide formulations, and combination therapies, are currently undergoing clinical evaluation and are expected to further transform obesity treatment. This review provides the current understanding of obesity pharmacotherapy, discusses approved anti-obesity medications, highlights emerging therapeutic agents and drugs under clinical development, and explores future perspectives in obesity management. Additionally, the review addresses current challenges, safety concerns, and public health strategies required to combat the growing obesity epidemic.

## Highlights

- Obesity is a chronic relapsing endocrine disease.
- GLP-1 receptor agonists have transformed obesity treatment.
- Tirzepatide produces >20% weight reduction.
- Triple agonists may approach bariatric surgery efficacy.
- Precision medicine will shape future obesity management.

## 1 Introduction

Obesity is a chronic, progressive, and relapsing metabolic disease resulting from excessive adiposity that impairs health and quality of life (1). According to the World Obesity Federation, obesity represents a major and growing global public health burden, with substantial variation in prevalence across regions and populations (2). As per the World Health Organization’s recommendation, adult obesity is identified as a BMI of at least 30 kg/m², whereas individuals with a BMI of 25.0-29.9 kg/m² are classified as overweight (3). However, for Asian populations, including Indians, lower BMI cut-offs are recommended because obesity-related metabolic complications occur at comparatively lower BMI values (4).

Obesity has become one of the fastest-growing non-communicable diseases worldwide (5). Sedentary lifestyles, urbanisation, unhealthy dietary habits, reduced physical activity, inadequate sleep, chronic stress, and genetic susceptibility have collectively contributed to the rapid increase in obesity prevalence (6). More than one billion people worldwide are living with obesity, with the burden continuing to rise across both developed and developing countries (5). India is also witnessing a substantial increase in obesity prevalence, particularly in urban populations, posing major challenges to healthcare systems due to the associated rise in diabetes, hypertension, cardiovascular disease, and chronic kidney disease (7).

Obesity is a complex disease involving dysregulation of multiple physiological pathways that control appetite, satiety, energy expenditure, and adipose tissue metabolism (8). The central nervous system, particularly the hypothalamus, integrates peripheral hormonal signals including leptin, ghrelin, insulin, GLP-1, glucose-dependent insulinotropic polypeptide (GIP), peptide YY (PYY), and cholecystokinin (CCK) to regulate food intake and body weight (9). Chronic overnutrition leads to leptin resistance, impaired satiety signalling, chronic low-grade inflammation, mitochondrial dysfunction, and alterations in the gut microbiome, all of which contribute to persistent weight gain and difficulty in maintaining long-term weight loss (10).

Individuals with obesity have a significantly greater likelihood of developing a wide range of comorbidities, such as type 2 diabetes mellitus, hypertension, dyslipidemia, coronary artery disease, stroke and metabolic dysfunction-associated with steatotic liver disease (MASLD), obstructive sleep apnea, infertility, osteoarthritis, depression, and several obesity-associated cancers (11). These complications contribute significantly to increased morbidity and mortality, increased healthcare expenditure, and reduced life expectancy (12).

Lifestyle modification comprising dietary intervention, increased physical activity, and behavioural therapy remains the foundation of obesity management (13). Nevertheless, compensatory neuroendocrine adaptations often lead to weight regain, limiting the long-term success of lifestyle measures alone (14). Consequently, pharmacotherapy has emerged as an important adjunct for individuals who do not achieve adequate weight reduction with lifestyle interventions or who have obesity-related comorbidities (13, 15).

The therapeutic landscape of obesity has changed dramatically during the past decade (8). Traditional anti-obesity drugs such as orlistat, phentermine/topiramate, and naltrexone/bupropion have been complemented by highly effective incretin-based therapies including liraglutide, semaglutide, and tirzepatide, which provide substantially greater and more durable weight reduction than earlier agents (16–18). Moreover, next-generation therapies such as retatrutide, CagriSema, amycretin, and orforglipron are undergoing advanced clinical development and may further improve treatment outcomes (19). These advances support the modern concept of obesity as a chronic disease requiring long-term, individualized, mechanism-based treatment rather than short-term weight loss interventions (1, 8).

The present review provides an overview of the pharmacological management of obesity, summarizes evidence on approved and investigational anti-obesity drugs, discusses ongoing clinical trials, evaluates safety considerations, and highlights future therapeutic strategies that may reshape obesity treatment in the coming years.

## 2 Literature search strategy

A comprehensive literature search was performed using PubMed, Scopus, Embase, Web of Science, ClinicalTrials.gov, and Google Scholar for studies published between January 2015 and June 2026. Search terms included “obesity”, “anti-obesity drugs”, “GLP-1 receptor agonists”, “tirzepatide”, “retatrutide”, “orforglipron”, “CagriSema”, “weight loss pharmacotherapy”, “clinical trial”, and related keywords. Preference was given to randomized controlled trials, systematic reviews, meta-analyses, international guidelines, and phase II/III clinical trials.

## 3 Pathophysiology of obesity

**Figure 1:**
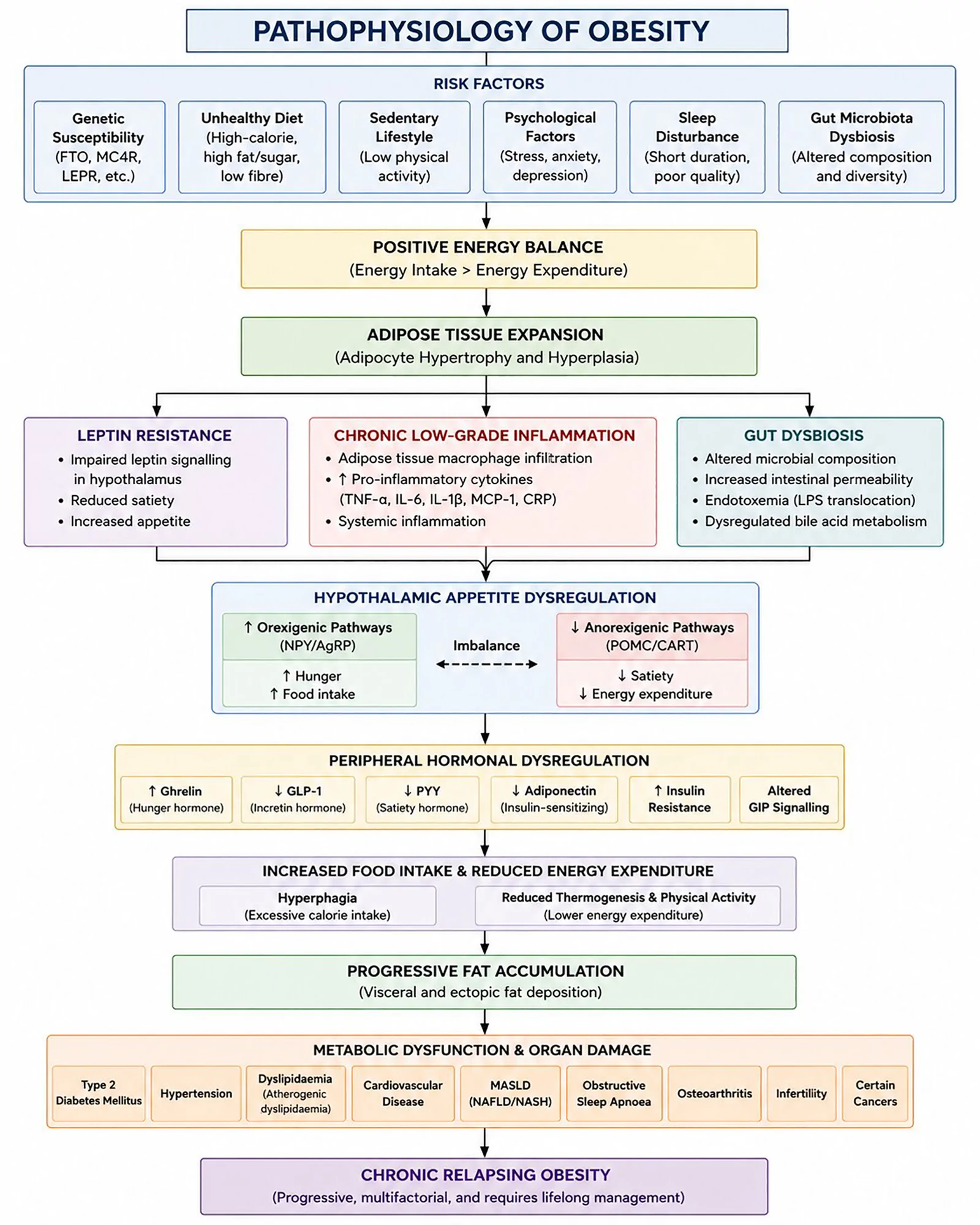
Pathophysiology of obesity. Schematic overview of the major mechanisms underlying obesity, illustrating the interaction among genetic susceptibility, environmental and lifestyle factors, hypothalamic appetite regulation, peripheral hormonal signalling, adipose tissue dysfunction, chronic inflammation, gut microbiota dysbiosis, and the gut–brain axis, culminating in obesity-related metabolic complications. AgRP, agouti-related peptide; CART, cocaine- and amphetamine-regulated transcript; CCK, cholecystokinin; CRP, C-reactive protein; GIP, glucose-dependent insulinotropic polypeptide; GLP-1, glucagon-like peptide-1; IL-6, interleukin-6; MCP-1, monocyte chemoattractant protein-1; NPY, neuropeptide Y; POMC, pro-opiomelanocortin; PYY, peptide YY; TNF-α, tumour necrosis factor-alpha.

Obesity is a chronic, multifactorial disease in which sustained positive energy balance represents a key underlying mechanism that interacts with genetic, environmental, behavioural, metabolic, endocrine, and hormonal factors to promote excessive adiposity (20). Although energy imbalance remains fundamental to obesity development, multiple biological and environmental determinants influence disease susceptibility and progression (21). The central nervous system, adipose tissue, gastrointestinal tract, pancreas, liver, skeletal muscle, and gut microbiota collectively regulate appetite, energy homeostasis, and body weight (22, 23). Dysregulation of these interconnected pathways promotes excessive adiposity and obesity-related metabolic complications (22) (**Figure 1**).

### 3.1 Energy homeostasis

Body weight is maintained through a dynamic balance between energy intake and energy expenditure (24). Total energy expenditure comprises basal metabolic rate, physical activity energy expenditure, and the thermic effect of food, with their relative contributions varying across individuals and the life course (25). Chronic positive energy balance leads to adipocyte hypertrophy and hyperplasia, resulting in progressive fat accumulation (20). Adipose tissue is recognized as an endocrine organ that secretes hormones and inflammatory mediators that influence glucose metabolism, insulin sensitivity, and systemic inflammation (20, 22). Energy expenditure comprises basal metabolic rate, the thermic effect of food, and physical activity energy expenditure, each contributing differently across the life course (25). Adaptive thermogenesis and alterations in energy expenditure following weight loss play important roles in long-term weight regain (26). Recent advances in indirect calorimetry, doubly labelled water methodology, and wearable technologies have substantially improved the assessment of human energy expenditure and metabolic adaptation (27, 28).

### 3.2 Central regulation of appetite

The hypothalamus is the primary regulator of appetite and energy balance (22). Within the arcuate nucleus, two opposing neuronal pathways regulate food intake (22, 29):

- Orexigenic neurons expressing neuropeptide Y (NPY) and agouti-related peptide (AgRP) stimulate hunger and reduce energy expenditure (29).
- Anorexigenic neurons expressing pro-opiomelanocortin (POMC) and cocaine- and amphetamine-regulated transcript (CART) suppress appetite and enhance satiety (29).

Peripheral metabolic signals continuously modulate these neuronal circuits to maintain energy homeostasis (22). In obesity, impaired responsiveness to these signals results in persistent hyperphagia and difficulty maintaining long-term weight loss (22).

Beyond the arcuate nucleus, additional hypothalamic nuclei, including the paraventricular nucleus (PVN), ventromedial hypothalamus (VMH), and dorsomedial hypothalamus (DMH), together with brainstem structures such as the nucleus tractus solitarius (NTS) and area postrema, integrate peripheral hormonal, nutrient, and vagal signals to coordinate appetite, satiety, and energy expenditure (29). Communication between these interconnected neuronal networks is essential for maintaining energy homeostasis, and alterations in these circuits may contribute to dysregulated feeding behaviour and obesity (22, 29). Increasing understanding of these neural pathways has also contributed to elucidating the central mechanisms through which incretin-based therapies, particularly GLP-1 receptor agonists, suppress appetite and promote weight loss (30); however, the precise neural sites and pathways responsible for their therapeutic effects remain an active area of investigation (30).

### 3.3 Peripheral hormonal regulation

Several gastrointestinal and adipose-derived hormones regulate appetite and metabolism (22).

Leptin, secreted by adipocytes, suppresses appetite by activating hypothalamic satiety pathways (29, 31). Despite elevated circulating leptin concentrations in obesity, hypothalamic leptin resistance develops, reducing satiety and promoting continued food intake (29, 32).

Ghrelin, produced primarily by the stomach, is a major orexigenic gastrointestinal hormone involved in appetite regulation (33). Ghrelin concentrations increase before meals and decline after food intake, contributing to meal initiation (33).

Insulin also contributes to central appetite regulation in addition to its metabolic actions (34, 35). Obesity-associated insulin resistance impairs both glucose homeostasis and hypothalamic satiety signalling (35).

Among gut-derived hormones, glucagon-like peptide-1 (GLP-1) plays a particularly important role by delaying gastric emptying, increasing satiety, suppressing glucagon secretion, and stimulating glucose-dependent insulin release (36). Alterations in GLP-1 and GIP signalling contribute to obesity and metabolic dysfunction, highlighting their central role in the physiological regulation of appetite and body weight (36, 37).

Other satiety hormones, including peptide YY (PYY) and cholecystokinin (CCK), further contribute to appetite suppression and meal termination through gut–brain communication (37).

### 3.4 Adipose tissue dysfunction and chronic inflammation

Adipose tissue is recognised as an endocrine organ that secretes numerous adipokines, including leptin, adiponectin, resistin, visfatin, chemerin, and omentin (38, 39). In obesity, adiponectin concentrations decrease, resulting in reduced insulin sensitivity and diminished anti-inflammatory activity (38, 39).

Hypertrophied adipocytes recruit macrophages and other immune cells into adipose tissue, leading to chronic low-grade inflammation characterised by increased production of tumour necrosis factor-alpha (TNF-α), interleukin-6 (IL-6), and monocyte chemoattractant protein-1 (MCP-1) (40, 41). These inflammatory mediators contribute to insulin resistance, endothelial dysfunction, atherosclerosis, and progression of metabolic disease (40, 41).

### 3.5 Gut microbiota and gut–brain axis

The intestinal microbiota plays a role in nutrient absorption, energy harvest, immune regulation, and metabolic homeostasis (42, 43). Alterations in gut microbial composition (dysbiosis) have been associated with increased energy extraction from food, impaired intestinal barrier function, chronic inflammation, and obesity (42, 43).

The gut-brain axis represents a complex bidirectional signalling system linking the gastrointestinal tract with the central nervous system through neuronal, hormonal and immune mechanisms (44, 45). Postprandial secretion of hormones such asGLP-1, GIP, PYY, and CCK play a key role in controlling food intake, satiety, and metabolic regulation (37, 44, 46). Many recently developed anti-obesity drugs exert their effects by enhancing signalling through this axis (30, 44).

### 3.6 Genetic and emerging mechanisms

Genetic susceptibility significantly influences the risk of obesity (47, 48). Variants in genes involved in appetite regulation and energy metabolism, including FTO, MC4R, LEPR, POMC, and PCSK1, contribute to interindividual variability in body weight (47, 48). Epigenetic modifications further influence gene expression in response to diet and physical activity (49).

Epigenetic mechanisms, including DNA methylation, histone modifications, and microRNA-mediated regulation, contribute to obesity by altering gene expression in response to nutritional, environmental, and lifestyle factors (49). These reversible molecular changes influence adipogenesis, inflammation, insulin sensitivity, and energy metabolism and have emerged as promising therapeutic targets for obesity prevention and treatment (49). Furthermore, advances in epigenetic research may facilitate precision medicine approaches by enabling individualized risk assessment and targeted therapeutic interventions (47, 49).

Emerging therapeutic strategies include activation of brown adipose tissue (BAT), induction of white adipose tissue browning, modulation of the gut microbiota, and manipulation of gut–brain signalling pathways (15, 50). Thermogenic adipose tissues have attracted considerable interest because their activation may increase energy expenditure and improve metabolic health (15, 50); however, the therapeutic relevance of BAT activation and white adipose tissue browning in humans remains under investigation (15, 50). Similarly, microbiota-directed interventions show promise, but substantial heterogeneity between individuals and limited causal and long-term clinical evidence remain important challenges (51).

## 4 Management of obesity

**Figure 2:**
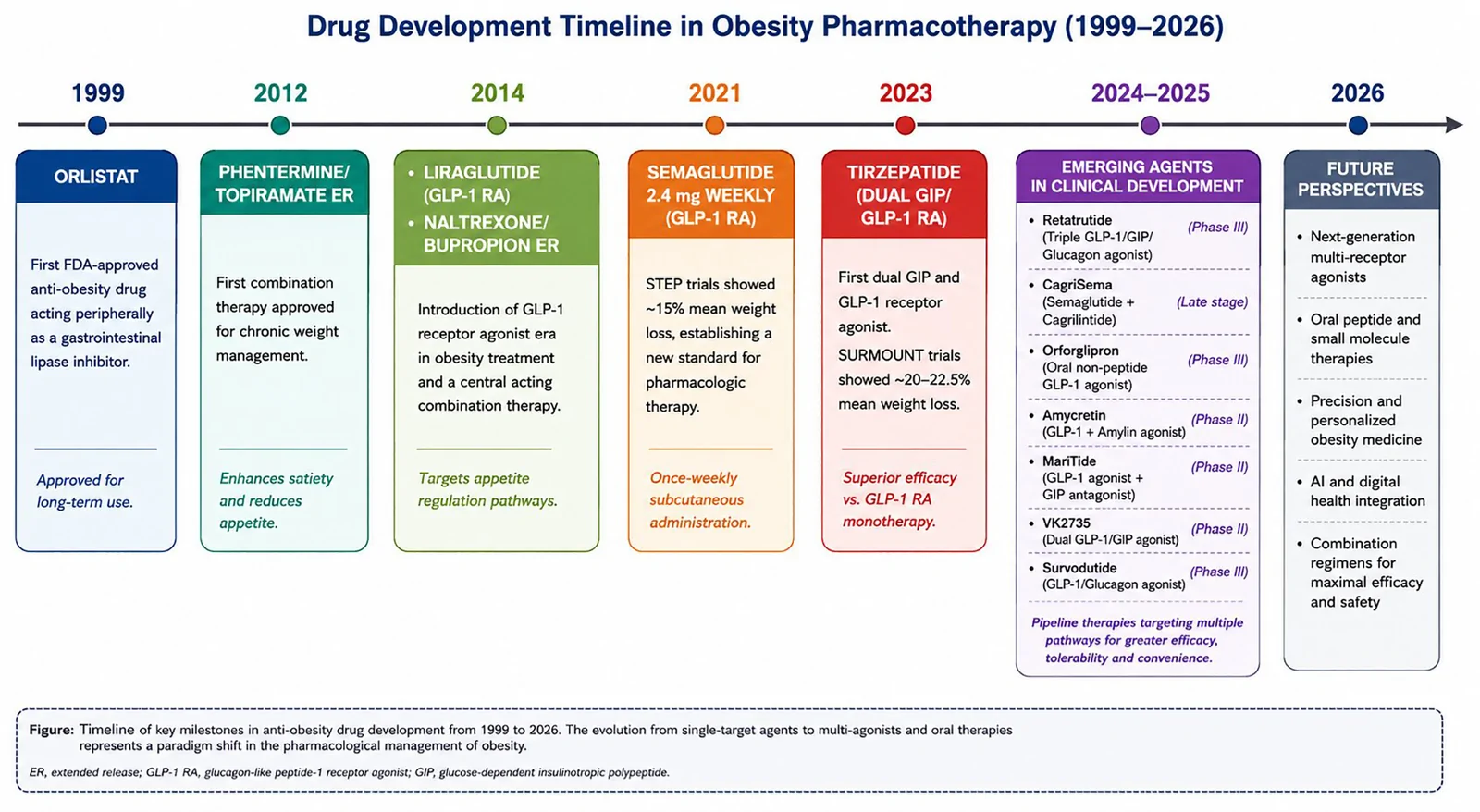
Comprehensive multidisciplinary approach to obesity management. Comprehensive management strategy for obesity integrating lifestyle modification, behavioural therapy, pharmacotherapy, bariatric surgery, and adjunctive herbal therapies to achieve sustained weight loss and improvement in cardiometabolic health.

Obesity management requires a comprehensive, patient-centred approach that integrates lifestyle modification, behavioural interventions, pharmacotherapy, and, where appropriate, metabolic surgery (13, 15) (**Figure 2**). Current international guidelines recommend individualised treatment based on body mass index (BMI), obesity-related comorbidities, previous treatment response, patient preferences, and long-term therapeutic goals (13, 15). While lifestyle intervention remains the cornerstone of management, pharmacological therapy is indicated for individuals who fail to achieve or sustain clinically meaningful weight loss through lifestyle measures (13, 15). Herbal formulations and nutraceuticals have also emerged as complementary approaches, although robust clinical evidence supporting their long-term efficacy remains limited alone (52, 53).

### 4.1 Non-pharmacological management

Lifestyle modification remains the first-line treatment for obesity and should continue throughout the course of therapy regardless of whether pharmacological or surgical interventions are initiated (13, 15).

#### 4.1.1 Dietary intervention

Dietary modification aims to achieve a sustained negative energy balance through individualised caloric restriction while maintaining nutritional adequacy (21, 54). Several dietary patterns have demonstrated effectiveness in reducing body weight and improving cardiometabolic health, including (53, 54):

- Mediterranean diet (54)
- DASH (Dietary Approaches to Stop Hypertension) diet (53)
- Low-carbohydrate diet (54)
- High-protein diet (54)
- Intermittent fasting (53)

No single dietary strategy has consistently demonstrated superiority over others; therefore, dietary plans should be individualised according to patient preferences, metabolic profile, and long-term adherence (54). Even a 5–10% weight loss improves glycaemic control, blood pressure, lipid profile, and overall cardiovascular risk (55).

#### 4.1.2 Physical activity

Regular physical activity increases energy expenditure and contributes to improvements in insulin sensitivity, physical fitness, and cardiometabolic health. Resistance training is particularly important for maintaining or improving muscle function during weight management (13, 56, 57).

Current recommendations include (13):

- 150–300 minutes/week of moderate-intensity aerobic physical activity
- ≥2 sessions/week of muscle-strengthening activities
- Reduction in prolonged sedentary behaviour

Exercise should be incorporated as a long-term component of obesity management and individualized according to physical capacity, comorbidities, treatment goals, and patient preference (13, 56, 57).

#### 4.1.3 Behavioural therapy

Behavioural interventions enhance adherence to dietary and exercise programmes by addressing unhealthy eating habits and sedentary lifestyles (58, 59).

Common behavioural strategies include (58, 59):

- Self-monitoring of diet and body weight (58)
- Goal setting (58)
- Cognitive behavioural therapy (CBT) (58, 59)
- Motivational interviewing (58)
- Stress management (58)
- Relapse prevention (58, 59)

Digital health applications and telemedicine increasingly support behavioural counselling and improve long-term treatment adherence (60, 61).

#### 4.1.4 Sleep and stress management

Sleep deprivation and chronic psychological stress contribute to obesity by disrupting appetite-regulating hormones such as leptin, ghrelin, and cortisol (62, 63). Improving sleep hygiene, practising stress-reduction techniques and mindfulness, and providing psychological support can enhance metabolic health and improve long-term weight management (62, 63).

#### 4.1.5 Bariatric and metabolic surgery

Metabolic surgery remains the most successful long-term therapeutic approach for severe obesity and is recommended for patients with (64, 65):

- BMI ≥40 kg/m², or (64)
- BMI ≥35 kg/m² with obesity-related comorbidities, particularly when conservative therapy has failed (64, 65).

Common surgical procedures include (64):

- Sleeve gastrectomy (64)
- Roux-en-Y gastric bypass (64)
- Adjustable gastric banding (64)
- Biliopancreatic diversion with duodenal switch (64)

These procedures produce substantial and durable weight loss while improving type 2 diabetes mellitus, hypertension, dyslipidaemia, and overall survival (64, 65). Lifelong nutritional supplementation and regular follow-up remain essential (64).

### 4.2 Herbal formulations in obesity management

Growing interest in herbal medicines and nutraceuticals has expanded their use as adjunctive therapies for obesity (66, 67). Plant-derived bioactive compounds exert anti-obesity effects through multiple mechanisms, including appetite suppression, inhibition of lipid absorption, enhancement of thermogenesis, modulation of adipogenesis, improvement of insulin sensitivity, regulation of gut microbiota, and reduction of oxidative stress and chronic inflammation (66–68) (**Table 1**).

**Table 1:** Major herbal agents with potential anti-obesity effects.

Herbal agent	Proposed mechanism
Green tea (Camellia sinensis) (69)	Thermogenesis, fat oxidation
Garcinia cambogia (70)	Appetite suppression, inhibition of lipogenesis
Glucomannan (71)	Increased satiety, delayed gastric emptying
Guggul (Commiphora mukul) (66, 72)	Lipid metabolism
Ginger (Zingiber officinale) (73)	Thermogenesis, anti-inflammatory effects
Fenugreek (*Trigonella foenum-graecum* L.) (74)	Appetite suppression, improved glycaemic control
Gymnema (*Gymnema sylvestre*) (75)	Reduced glucose absorption
Curcumin (derived from *Curcuma longa* L.) (76)	Anti-inflammatory and antioxidant effects
Capsaicin (derived from *Capsicum annuum* L.) (77)	Increased energy expenditure
Berberine (derived from *Berberis* spp.) (78)	Improved insulin sensitivity
Ashwagandha (*Withania somnifera* (L.) Dunal) (79)	Stress reduction and metabolic regulation
Triphala (80)	Gastrointestinal and metabolic effects

Representative medicinal plants and phytochemicals with reported anti-obesity activities and their proposed mechanisms of action based on experimental and clinical evidence. Clinical efficacy varies among formulations, and further randomised controlled trials are required to establish long-term safety and effectiveness.

Unlike conventional pharmacotherapy, herbal formulations contain multiple phytochemicals that may act synergistically on several metabolic pathways (66, 67). However, considerable variability in composition, standardisation, and quality control limits their widespread clinical application (66, 67).

Polyherbal formulations combine several medicinal plants to produce synergistic effects on appetite regulation, glucose homeostasis, and lipid metabolism (66, 72). Nutraceuticals such as dietary fibres, probiotics, prebiotics, omega-3 fatty acids, and plant-derived bioactive compounds may further enhance weight management by improving gut microbiota composition and metabolic health (52, 53, 67).

Despite encouraging findings from preclinical studies and selected clinical trials, the overall quality of evidence remains limited (66, 67). Standardised formulations, well-designed randomised controlled trials, and long-term safety evaluations are required before herbal therapies can be routinely recommended in clinical practice (66, 67, 72).

### 4.3 Currently approved anti-obesity drugs

**Figure 3:**
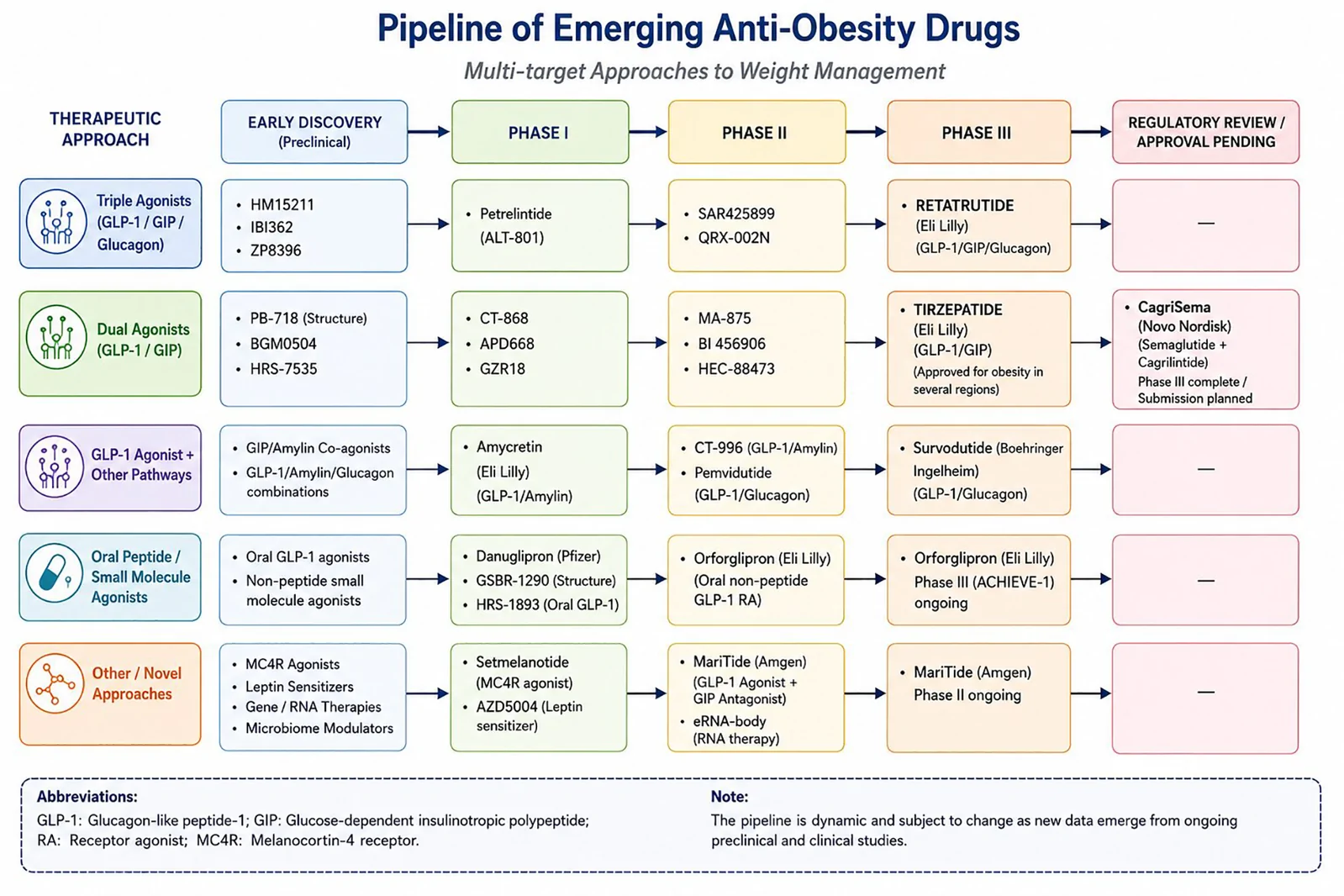
Mechanisms of action of currently approved anti-obesity drugs. Mechanisms of action of currently approved anti-obesity medications. Orlistat decreases intestinal fat absorption by inhibiting pancreatic lipase; centrally acting agents suppress appetite, whereas incretin-based therapies enhance satiety, delay gastric emptying, and improve glucose homeostasis. GIP, glucose-dependent insulinotropic polypeptide; GLP-1, glucagon-like peptide-1.

**Figure 4:**
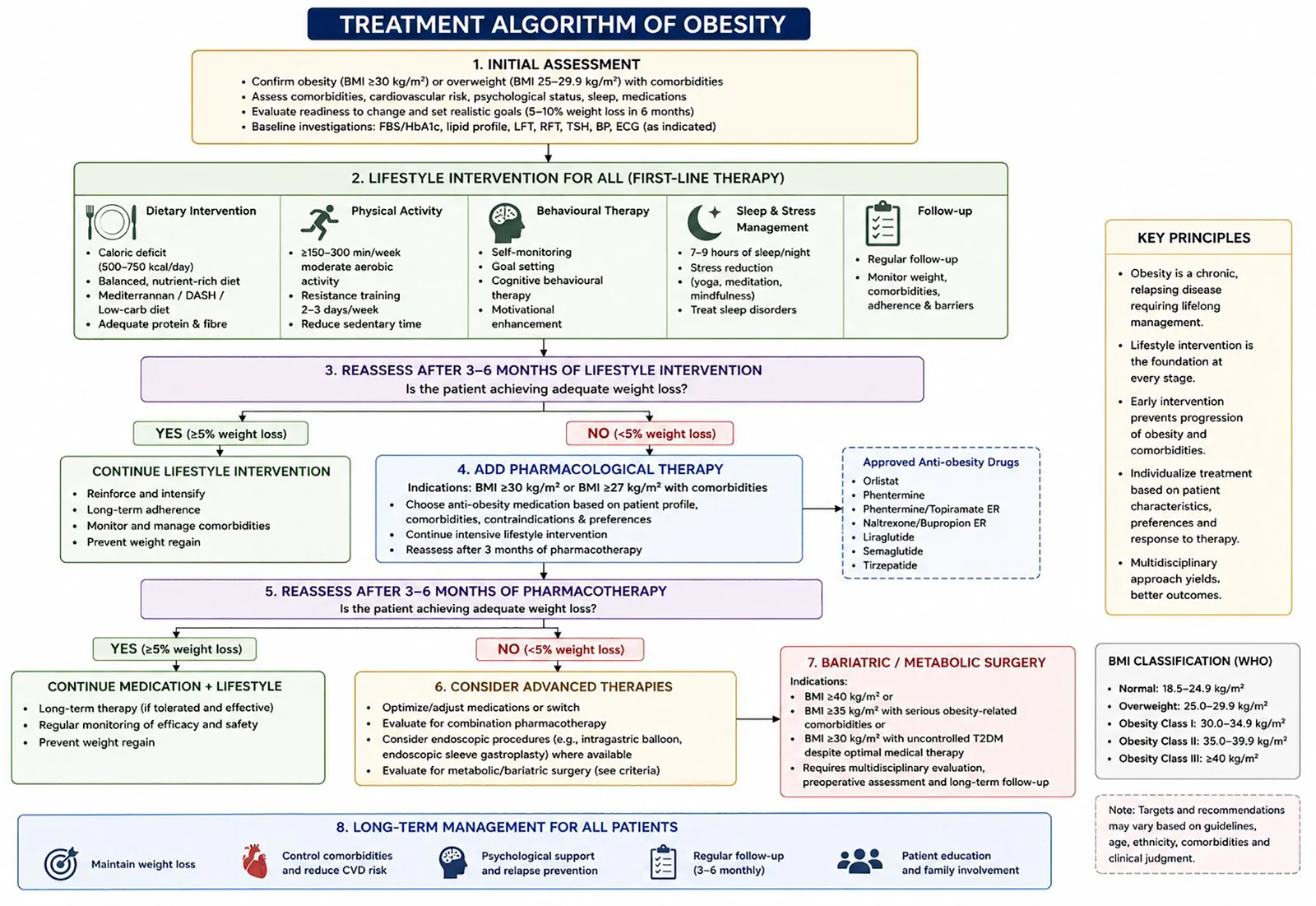
Drug development timeline in obesity pharmacotherapy (1999–2026). Timeline highlighting major milestones in the development of anti-obesity pharmacotherapy from first-generation agents to incretin-based therapies and emerging multi-receptor agonists. The evolution reflects the transition from single-target pharmacological approaches to highly effective multi-target therapies with improved weight-loss efficacy. ER, extended release; FDA, U.S. Food and Drug Administration; GIP, glucose-dependent insulinotropic polypeptide; GLP-1 RA, glucagon-like peptide-1 receptor agonist.

Pharmacotherapy is an adjunct to lifestyle modification for adults with a body mass index (BMI) ≥30 kg/m² or ≥27 kg/m² in the presence of comorbidities such as type 2 diabetes mellitus, hypertension, dyslipidaemia, obstructive sleep apnoea, or cardiovascular disease (13, 15). Anti-obesity medications facilitate weight reduction by suppressing appetite, enhancing satiety, reducing intestinal fat absorption, or improving energy homeostasis (81) (**Figure 3**). Drug selection should be individualised based on efficacy, safety, comorbidities, patient preference, route of administration, and cost (13, 15) (**Table 2**). The therapeutic importance of incretin biology has transformed the pharmacological management of obesity (36). GLP-1 receptor agonists have demonstrated substantial and sustained weight reduction by enhancing satiety, delaying gastric emptying, and reducing energy intake (36). More recently, dual GIP/GLP-1 receptor agonists, such as tirzepatide, have shown greater efficacy than GLP-1 receptor agonists alone, producing substantial weight loss in clinical trials (18, 82) (**Figure 4**). These advances have established incretin-based therapies as a cornerstone of modern obesity pharmacotherapy (15).

**Table 2:** Comparison of currently approved anti-obesity drugs.

Drug	Mechanism	Route	Mean weight loss	Major adverse effects
Orlistat	Pancreatic lipase inhibitor	Oral	5–8% (83)	Steatorrhoea, oily stools
Phentermine	Sympathomimetic appetite suppressant	Oral	5–10% (84)	Tachycardia, insomnia
Phentermine/Topiramate ER	Appetite suppression + enhanced satiety	Oral	8–10% (85, 86)	Dry mouth, paraesthesia, teratogenicity
Naltrexone/Bupropion ER	Central appetite regulation	Oral	5–9% (89, 90)	Nausea, hypertension, seizure risk
Liraglutide	GLP-1 receptor agonist	SC daily	8–10% (91, 92)	Gastrointestinal adverse effects
Semaglutide	GLP-1 receptor agonist	SC weekly	~15% (17, 93)	Gastrointestinal adverse effects
Tirzepatide	Dual GIP/GLP-1 receptor agonist	SC weekly	~20% (18, 97)	Gastrointestinal adverse effects

Comparison of currently approved anti-obesity medications according to mechanism of action, route of administration, average weight loss, and major adverse effects. Weight-loss values are approximate averages reported in pivotal clinical trials and may vary depending on study population and treatment duration.

ER, extended release; GIP, glucose-dependent insulinotropic polypeptide; GLP-1, glucagon-like peptide-1; SC, subcutaneous.

#### 4.3.1 Orlistat

The anti-obesity effect of orlistat results from inhibition of gastrointestinal lipases, which limits the breakdown and subsequent absorption of dietary triglycerides (16, 83). Because its action is confined primarily to the gastrointestinal tract, systemic exposure is minimal (83).

The recommended dose is 120 mg orally three times daily with fat-containing meals (83). When combined with lifestyle modification, orlistat produces an average weight reduction of 5–8% over one year (16, 83). Additional benefits include modest improvements in lipid profile and decreased blood sugar level in high-risk individuals (16, 83).

Long-term clinical studies have demonstrated that 52-week administration of orlistat significantly reduces visceral adiposity while maintaining an acceptable safety profile (16, 83). Sustained treatment combined with lifestyle modification contributes to durable weight loss and improvements in cardiometabolic risk factors (16, 83).

Treatment with orlistat commonly causes gastrointestinal side effects, such as oily stools, steatorrhoea, flatulence, faecal urgency, and abdominal discomfort due to reduced fat absorption (83).

Orlistat is contraindicated in patients with chronic malabsorption syndrome, cholestasis, pregnancy, or hypersensitivity to the drug (83).

#### 4.3.2 Phentermine

Phentermine is a sympathomimetic amine that decreases appetite by increasing hypothalamic norepinephrine release (84). It is approved primarily for short-term management of obesity (84).

The usual dose ranges from 15–37.5 mg once daily, preferably in the morning (84). Clinical studies report average weight reductions of 5–10% over three to six months (84).

Common adverse effects include insomnia, dry mouth, tachycardia, palpitations, constipation, anxiety, and increased blood pressure (84). Because of its sympathomimetic activity, phentermine should not be given in patients with uncontrolled hypertension, cardiovascular disease, hyperthyroidism, glaucoma, pregnancy, or concurrent monoamine oxidase inhibitor therapy (84).

#### 4.3.3 Phentermine/topiramate extended release

Phentermine/topiramate extended- release combines the appetite suppression of phentermine with enhanced satiety produced by topiramate, resulting in greater efficacy than either agent alone (85).

Treatment is initiated at 3.75 mg/23 mg once daily, followed by dose escalation according to clinical response (85). Large randomised clinical trials have demonstrated average weight reductions of 8–10% after one year of therapy, together with improvements in blood pressure, lipid profile, and glycaemic control (85, 86).

Recent randomized clinical trials have further demonstrated the effectiveness of phentermine/topiramate extended-release in individuals showing an inadequate response to behavioural interventions (86). Emerging evidence also suggests that predictors of treatment response, including baseline clinical characteristics and metabolic profiles, may facilitate individualized obesity management, particularly among adolescents and other high-risk populations (87, 88).

Frequently reported adverse effects include dry mouth, paraesthesia, constipation, insomnia, dizziness, and dysgeusia (85, 86). Topiramate is teratogenic; therefore, effective contraception is mandatory in women of childbearing potential (85). The combination should not be used in pregnant mothers and should be prescribed cautiously in patients with cardiovascular disease (85).

#### 4.3.4 Naltrexone/bupropion extended release

Naltrexone/bupropion combines an opioid receptor antagonist with a norepinephrine–dopamine reuptake inhibitor to enhance hypothalamic satiety signalling and reduce food cravings (89).

The target maintenance dose is 32 mg naltrexone/360 mg bupropion daily, achieved by gradual weekly dose escalation (89).

Clinical trials have demonstrated average weight reductions of 5–9% over one year, accompanied by improvements in waist circumference and insulin sensitivity (89, 90).

Observable side effects include nausea, vomiting, constipation, headache, dizziness, dry mouth, and insomnia (89, 90). Serious concerns include increased blood pressure, seizure risk, and neuropsychiatric adverse events (89). The combination is contraindicated in patients with uncontrolled hypertension, seizure disorders, eating disorders, chronic opioid therapy, or pregnancy (89).

#### 4.3.5 Liraglutide

Liraglutide is a long-acting GLP-1 receptor agonist administered once daily by subcutaneous injection (91). It promotes weight loss by enhancing satiety, delaying gastric emptying, reducing appetite, stimulating glucose-dependent insulin secretion, and suppressing glucagon release (36, 91).

Treatment begins with 0.6 mg daily, gradually increasing to a maintenance dose of 3 mg/day (91).

The SCALE clinical trial programme demonstrated average weight reductions of 8–10% after 56 weeks, together with improvements in glycaemic control, blood pressure, and cardiovascular risk factors (91, 92).

The commonly reported side effects are nausea, vomiting, diarrhoea, constipation, and gallbladder disease (91, 92). Rare but important adverse events include pancreatitis (91). Liraglutide should not be given in patients with a history of thyroid carcinoma, multiple endocrine neoplasia syndrome type 2 (MEN2), or pregnancy (91).

#### 4.3.6 Semaglutide

Semaglutide is a second-generation long-acting GLP-1 receptor agonist administered once weekly by subcutaneous injection (17). Compared with liraglutide, semaglutide produces greater appetite suppression and more sustained weight reduction (17, 93).

The recommended maintenance dose for obesity is 2.4 mg once weekly, following gradual dose escalation over approximately 16–20 weeks (17).

The STEP clinical trial programme demonstrated substantial weight reduction with semaglutide, with a mean weight loss of approximately 15% after 68 weeks of treatment (17, 93). In addition to weight reduction, semaglutide improves glycaemic control, blood pressure, lipid profile, inflammatory markers, and quality of life (17, 93). Cardiovascular outcome studies have also demonstrated reductions in cardiovascular events in patients with obesity and already have cardiovascular disease (94).

Adverse effects are primarily gastrointestinal and include nausea, vomiting, diarrhoea, constipation, and gallstones (17, 93). Rare cases of pancreatitis have also been reported (17). Similar to liraglutide, semaglutide should be avoided in patients with medullary thyroid carcinoma, MEN2, or pregnancy (17, 91).

Recent phase III evidence has demonstrated clinically meaningful weight reduction with once-daily oral semaglutide 25 mg in adults with overweight or obesity (95). In the OASIS 4 trial, oral semaglutide 25 mg produced a mean weight reduction of 13.6% at week 64, compared with 2.2% with placebo, providing a potential oral alternative to injectable semaglutide therapy (95).

Although semaglutide produces substantial weight reduction, its gastrointestinal tolerability, need for continued treatment, and relatively high cost may influence treatment persistence in routine clinical practice (93, 96).

#### 4.3.7 Tirzepatide

Tirzepatide is the first approved dual GIP/GLP-1 receptor agonist and represents a major advance in obesity pharmacotherapy (18, 82). Simultaneous activation of GLP-1 and GIP receptors enhances satiety, delays gastric emptying, improves insulin sensitivity, reduces caloric intake, and produces greater weight reduction than GLP-1 receptor agonists alone (18, 82).

Treatment is initiated with 2.5 mg once weekly and gradually escalated to 5, 10, or 15 mg weekly, depending on clinical response and tolerability (18).

In the SURMOUNT-5 trial, tirzepatide produced a least-squares mean weight reduction of 20.2% at 72 weeks, compared with 13.7% with semaglutide, demonstrating superior weight reduction with tirzepatide in adults with obesity without type 2 diabetes (97). Tirzepatide also significantly improves glycaemic control, waist circumference, blood pressure, lipid profile, and markers of metabolic dysfunction-associated steatotic liver disease (18, 97).

The adverse effects most commonly observed are gastrointestinal, including nausea, vomiting, diarrhoea, and constipation (18, 82). Gallbladder disease and pancreatitis are uncommon but clinically important adverse events (18). Contraindications are similar to those of GLP-1 receptor agonists (18).

The greater mean weight reduction observed with tirzepatide compared with semaglutide represents an important therapeutic advance; however, interpretation should consider that direct head-to-head evidence is more informative than cross-trial comparisons, and long-term comparative safety and treatment persistence remain important considerations (82, 96).

### 4.4 Critical considerations in current anti-obesity pharmacotherapy

Although currently approved anti-obesity medications have substantially expanded therapeutic options, their clinical effects and applicability vary considerably (8, 15). Older agents such as orlistat and centrally acting sympathomimetic therapies generally produce more modest weight reduction than newer incretin-based treatments, but their lower cost, oral administration, or established safety experience may remain relevant in selected patients (15, 83, 84). Conversely, GLP-1 receptor agonists and dual GIP/GLP-1 receptor agonists provide greater average weight reduction but are frequently associated with gastrointestinal adverse effects, higher treatment costs, and limitations related to injectable administration (17, 18, 96). Importantly, differences in reported weight loss should not be interpreted as direct evidence of superiority unless therapies have been evaluated in adequately powered head-to-head trials, because clinical trials differ in population characteristics, treatment duration, background lifestyle interventions, adherence, and outcome definitions (82, 96).

Furthermore, obesity is a chronic and relapsing disease, and pharmacological treatment generally requires prolonged therapy to maintain weight reduction (1, 14). Evidence of substantial weight regain following treatment discontinuation indicates that pharmacotherapy should not be considered a short-term intervention for most patients (14, 98). Therefore, treatment selection should consider not only the magnitude of weight loss but also tolerability, comorbidities, treatment persistence, affordability, route of administration, patient preference, and the feasibility of long-term follow-up (13, 15). These considerations support an individualized approach rather than selection of therapy solely according to the greatest mean percentage weight reduction reported in clinical trials (13, 15).

## 5 Emerging anti-obesity drugs and drugs under clinical development

**Figure 5:**
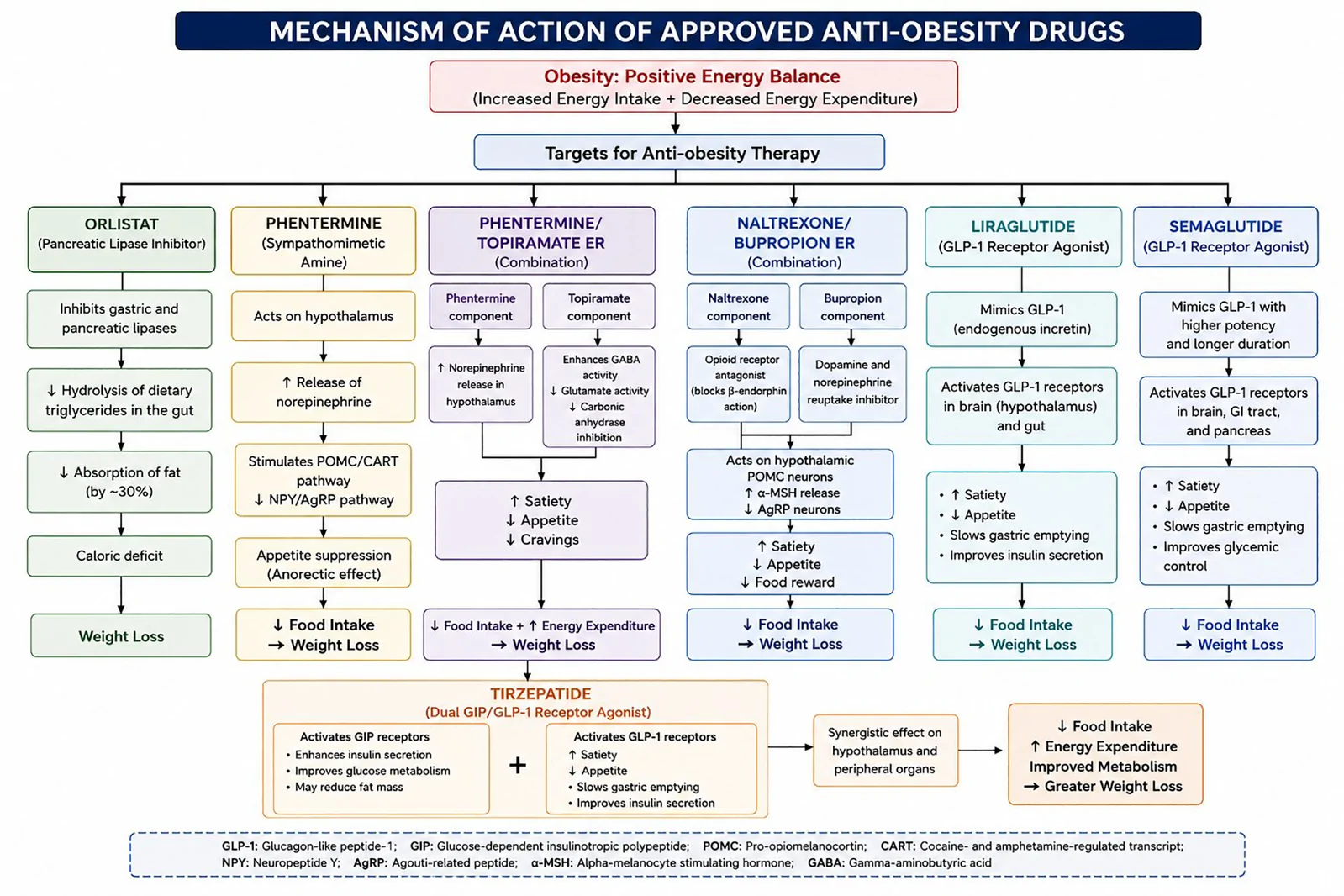
Pipeline of emerging anti-obesity drugs under clinical development. Current pipeline of investigational anti-obesity drugs according to therapeutic target and stage of clinical development. Emerging therapies primarily target GLP-1, GIP, glucagon, amylin, and other metabolic pathways to improve weight reduction, metabolic control, and long-term treatment outcomes. GIP, glucose-dependent insulinotropic polypeptide; GLP-1, glucagon-like peptide-1; MC4R, melanocortin-4 receptor; RA, receptor agonist.

Recent advances in obesity pharmacotherapy have shifted the focus from single-target therapies to multi-receptor agonists that simultaneously regulate appetite, satiety, glucose metabolism, and energy expenditure (8, 19, 99). Although semaglutide and tirzepatide have substantially improved treatment outcomes, many patients continue to experience residual obesity, weight regain after treatment discontinuation, or intolerance to injectable therapies (14, 96, 98). Consequently, several novel pharmacological agents are undergoing clinical development to achieve greater efficacy, improved tolerability, convenient oral administration, and better long-term adherence (19, 100) (**Figure 5**; **Table 3**).

**Table 3:** Emerging anti-obesity drugs under clinical development.

Drug	Target	Route	Clinical stage	Expected/reported weight loss
Retatrutide	GLP-1/GIP/Glucagon	SC weekly	Phase III	28.3% at 80 weeks (101)
Orforglipron	GLP-1 receptor	Oral once daily	Phase III	~14.7% at 36 weeks (103, 104)
CagriSema	GLP-1 + Amylin	SC weekly	Phase III	20.4% at 68 weeks; 23.0% at 84 weeks in REDEFINE 4 (105–107)
Amycretin (zenagamtide)	GLP-1 + Amylin	SC/Oral	Phase III	Up to ~24.3% at 36 weeks in phase 1b/2a clinical studies (108)
MariTide	GLP-1 agonist + GIP antagonist	SC	Phase III	12.3–16.2% weight loss at 52 weeks in phase II study (109)
VK2735	GLP-1/GIP	SC/Oral	Phase II	Up to 14.7% at 13 weeks (110)
Survodutide	GLP-1/Glucagon	SC weekly	Phase III	Significant weight reduction demonstrated in Phase III (111, 112)
Mazdutide	GLP-1/Glucagon	SC weekly	Phase III	Up to ~18.5% at 60 weeks (113)
GSBR-1290 (aleniglipron)	GLP-1 receptor	Oral	Phase IIb	Promising weight-loss efficacy in Phase II studies (114)
CT-388	GLP-1/GIP	SC weekly	Phase II	Clinically meaningful weight reduction demonstrated in Phase II (115)
ECC5004	Oral GLP-1 receptor agonist	Oral	Phase II	Promising early weight-loss efficacy (116)

Summary of selected investigational anti-obesity drugs, their molecular targets, routes of administration, clinical development stages, and reported weight-loss efficacy. Clinical development stages and efficacy estimates reflect evidence available up to June 30, 2026. Weight-loss estimates are derived from individual clinical trials and should not be interpreted as direct comparative measures of efficacy because study populations, treatment duration, background lifestyle intervention, adherence, and statistical estimands differ. Development status is subject to change as additional clinical-trial results and regulatory decisions become available.

GIP, glucose-dependent insulinotropic polypeptide; GLP-1, glucagon-like peptide-1; SC, subcutaneous.

### 5.1 Retatrutide

Retatrutide is an investigational first-in-class triple GIP/GLP-1/glucagon receptor agonist that simultaneously activates the GIP, GLP-1, and glucagon receptors (19, 100, 101). This multi-receptor mechanism is intended to suppress appetite, enhance satiety, improve metabolic control, and increase energy expenditure (19, 100).

Early phase II studies demonstrated substantial weight reduction, with mean weight loss of approximately 24% at 48 weeks. Subsequently, phase III evidence from the TRIUMPH-1 trial demonstrated substantially greater weight reduction, with participants receiving retatrutide 12 mg achieving a mean weight loss of 28.3% at 80 weeks. Retatrutide has demonstrated among the largest mean weight reductions reported to date in phase III obesity trials; however, it remains investigational and has not received regulatory approval as of June 2026 (19, 100–102).

Despite its high efficacy, interpretation should consider that long-term safety, treatment persistence, cardiovascular outcomes, and real-world effectiveness remain important areas of ongoing investigation.

### 5.2 Orforglipron

Orforglipron is an oral, non-peptide GLP-1 receptor agonist being developed for the treatment of obesity and type 2 diabetes. Its once-daily oral administration without the fasting requirements associated with some oral peptide formulations may provide a convenient alternative to injectable incretin therapies (103, 104).

Phase II clinical studies demonstrated dose-dependent weight reduction, with mean weight loss reaching approximately 14.7% at 36 weeks at the highest dose evaluated. Subsequent phase III studies have further evaluated its efficacy, safety, and maintenance of weight reduction in adults with obesity (103, 104).

As of June 2026, orforglipron remained an investigational therapy in clinical development. Its potential advantages include oral administration and the possibility of improved treatment convenience; however, long-term comparative effectiveness, tolerability, treatment persistence, and cost-effectiveness require continued evaluation.

### 5.3 CagriSema

CagriSema is a fixed-dose combination of cagrilintide, a long-acting amylin analogue, and semaglutide, a GLP-1 receptor agonist. The combination targets complementary pathways involved in appetite regulation, satiety, gastric emptying, and energy intake (105, 106).

In the phase III REDEFINE 1 trial, CagriSema produced a mean weight reduction of 20.4% at 68 weeks under the treatment-policy estimand, which reflects the treatment effect regardless of treatment discontinuation or other intercurrent events. Under the estimand assessing the effect if participants adhered to the assigned treatment, the corresponding mean weight reduction was 22.7% (105). More recently, the phase III REDEFINE 4 trial compared CagriSema with tirzepatide 15 mg in adults with overweight or obesity and demonstrated a mean weight reduction of 23.0% with CagriSema versus 25.5% with tirzepatide at 84 weeks under the efficacy estimand. Under the treatment-regimen estimand, which reflects the effect of treatment as assigned while accounting for treatment discontinuation and other intercurrent events, weight reduction was 20.2% with CagriSema versus 23.6% with tirzepatide. Importantly, CagriSema did not meet the prespecified criterion for non-inferiority to tirzepatide for weight reduction in the primary analysis (107). These findings demonstrate substantial efficacy of CagriSema but also highlight the importance of distinguishing estimands and avoiding direct comparison of weight-loss estimates without considering treatment adherence, discontinuation, and trial design (105, 107).

These findings indicate substantial weight-loss efficacy; however, differences between treatment estimands and head-to-head trial results highlight the importance of interpreting weight-loss estimates within the context of trial design, adherence, treatment duration, and comparator therapy.

### 5.4 Amycretin

Amycretin (zenagamtide) is an investigational unimolecular GLP-1 and amylin receptor agonist developed in both subcutaneous and oral formulations. Early clinical studies have demonstrated substantial weight-reduction potential. In a phase 1b/2a study of adults with overweight or obesity without diabetes, once-weekly subcutaneous amycretin produced mean weight reductions of up to 24.3% at 36 weeks at the highest dose evaluated, compared with approximately 1.1% with placebo (108). Oral amycretin has also demonstrated clinically meaningful weight reduction in early-phase studies, although the magnitude of weight loss was lower over the shorter treatment duration (108). Based on completed clinical studies and regulatory feedback, development of both subcutaneous and oral amycretin for weight management advanced to phase III in 2026. Therefore, amycretin should currently be regarded as an investigational therapy in phase III clinical development rather than as a phase II agent (108).

### 5.5 MariTide

MariTide (maridebart cafraglutide) is a long-acting investigational peptide–antibody conjugate that combines GLP-1 receptor agonism with GIP receptor antagonism, representing a distinct approach to incretin-based obesity pharmacotherapy (109). In a phase II randomized, placebo-controlled trial, once-monthly maridebart cafraglutide produced substantial weight reduction in adults with obesity, with mean weight loss at 52 weeks ranging from 12.3% to 16.2% across treatment groups compared with 2.5% with placebo under the treatment-policy estimand. In participants with obesity and type 2 diabetes, mean weight reduction ranged from 8.4% to 12.3% compared with 1.7% with placebo (109). Gastrointestinal adverse events were common, although no unexpected safety signals were identified in the phase II study. MariTide has subsequently advanced to phase III development in the MARITIME clinical trial programme, which is evaluating its efficacy and safety in obesity and obesity-related conditions, including chronic weight management, type 2 diabetes, cardiovascular disease, heart failure, and obstructive sleep apnea. Therefore, as of 2026, MariTide should be described as an investigational therapy in phase III clinical development rather than as a phase II agent (109).

### 5.6 Other emerging therapies

Several additional investigational agents are currently undergoing clinical evaluation, including:

- VK2735: VK2735 is an investigational dual GLP-1/GIP receptor agonist being developed in both subcutaneous and oral formulations. In the phase II VENTURE study, weekly subcutaneous VK2735 produced dose-dependent weight reduction, with the highest dose resulting in a mean weight reduction of 14.7% after 13 weeks (110). Phase II evaluation of the oral formulation has also demonstrated clinically meaningful weight reduction, supporting further development of VK2735 as a potentially convenient oral or injectable treatment option. As of June 2026, VK2735 remained in phase II clinical development (110).
- Survodutide – Survodutide is an investigational dual GLP-1/glucagon receptor agonist designed to combine the appetite-suppressing and metabolic effects of GLP-1 receptor activation with the potential energy-expenditure and metabolic effects of glucagon receptor activation (111). Survodutide demonstrated substantial weight reduction in phase II studies and subsequently progressed to phase III evaluation. The phase III SYNCHRONIZE-1 trial demonstrated significantly greater weight reduction with survodutide than placebo in adults with obesity without diabetes, supporting continued clinical development (111, 112). Despite these promising findings, longer-term data are required to establish its comparative efficacy, safety, treatment persistence, and effects on obesity-related complications.
- Mazdutide (113)
- GSBR-1290 (114)
- CT-388 (115)
- ECC5004 (116)

### 5.7 Critical appraisal of emerging therapies

Future therapeutic strategies increasingly focus on combination therapies that target multiple hormonal pathways involved in appetite regulation and energy metabolism (8, 19, 100).

The rapid development of multi-receptor agonists and combination therapies represents a major shift in obesity pharmacotherapy; however, the impressive weight reductions reported in early-phase trials should be interpreted cautiously (19, 100). Many emerging agents have been evaluated primarily in phase I or phase II studies with relatively short follow-up periods, and their long-term safety, treatment persistence, cardiovascular effects, and effects on obesity-related complications remain incompletely characterized (96, 100). In addition, cross-trial comparisons of weight loss may be misleading because studies differ in baseline body weight, diabetes status, treatment duration, dose-escalation schedules, background lifestyle intervention, and statistical methods (82, 96). The efficacy observed in highly selected clinical-trial populations may also differ from outcomes achieved in routine clinical practice (96).

Another important uncertainty concerns the sustainability of treatment effects (14, 98). As obesity is a chronic disease, the clinical value of a therapy depends not only on initial weight reduction but also on the ability to maintain clinically meaningful weight loss over many years (1, 14). The high cost of newer therapies, variable availability across countries, potential supply limitations, gastrointestinal adverse effects, and the need for long-term treatment may restrict their real-world accessibility (96). Consequently, the future success of emerging agents should be evaluated not solely by percentage weight loss, but through a broader assessment incorporating durability, safety, cardiometabolic outcomes, quality of life, adherence, cost-effectiveness, and equitable access (13, 15, 96).

## 6 Comparative analysis of current and emerging anti-obesity drugs

The therapeutic landscape of obesity has changed substantially over the past decade, with a transition from predominantly modest-efficacy therapies toward highly effective incretin-based and multi-receptor pharmacotherapies (117, 118) (**Table 4**). However, comparison of anti-obesity medications based solely on the magnitude of reported weight loss can be misleading. The percentage weight reduction observed in individual trials is influenced by baseline BMI, diabetes status, treatment duration, dose escalation, adherence, concomitant lifestyle intervention, and the characteristics of the enrolled population. Therefore, efficacy estimates from separate clinical trials should not automatically be interpreted as evidence of therapeutic superiority.

**Table 4:** Comparison of currently approved and emerging anti-obesity drugs.

Drug	Mean weight loss	Major clinical benefit	Limitation
Orlistat	5–8% (83)	Low systemic toxicity	Gastrointestinal adverse effects
Phentermine	5–10% (84)	Rapid appetite suppression	Short-term use; cardiovascular stimulation
Phentermine/Topiramate ER	8–10% (85, 86)	Greater efficacy than monotherapy	Teratogenicity
Naltrexone/Bupropion ER	5–9% (89, 90)	Reduces food cravings	Neuropsychiatric adverse effects
Liraglutide	8–10% (91, 92)	Improved glycaemic control	Daily injection
Semaglutide	~15% (17, 94)	Proven cardiovascular benefit	Gastrointestinal adverse effects
Tirzepatide	20.2% (18, 97)	Greater weight reduction than semaglutide in a head-to-head trial	High cost
Retatrutide*	28.3% at 80 weeks (101)	Triple receptor agonism	Investigational

Comparison of approved and selected investigational anti-obesity drugs with respect to average weight-loss efficacy, principal clinical benefits, and major limitations. Investigational agents have not yet received regulatory approval for routine clinical use. The symbol “*” indicates that retatrutide is an investigational drug and has not yet received regulatory approval for routine clinical use.

Head-to-head evidence provides a more informative basis for comparative assessment. For example, direct comparison of tirzepatide and semaglutide demonstrated greater weight reduction with tirzepatide; however, the clinical significance of differences in mean weight loss must be considered alongside tolerability, treatment persistence, comorbidity profile, route of administration, cost, and patient preference (97, 118, 119). Similarly, although newer therapies can produce substantially greater weight reduction than older agents, higher efficacy does not necessarily translate into better population-level effectiveness if treatment access, affordability, adherence, or long-term persistence is limited (117, 120).

Importantly, weight reduction should not be considered the sole outcome when evaluating anti-obesity medications. Improvements in glycaemic control, blood pressure, lipid abnormalities, cardiovascular outcomes, hepatic disease, quality of life, physical functioning, and obesity-related complications are also clinically relevant (120, 121). Conversely, adverse effects, treatment discontinuation, contraindications, drug interactions, and long-term safety must be incorporated into therapeutic decision-making (117, 121). Thus, the optimal anti-obesity medication is not necessarily the drug associated with the greatest mean weight loss, but the therapy that provides an appropriate balance between efficacy, safety, durability, accessibility, and patient-specific treatment goals (120).

Selection of an anti-obesity medication should be individualised according to the degree of obesity, associated comorbidities, expected efficacy, safety profile, route of administration, patient preference, accessibility, and cost (120). Lifestyle modification should remain an integral component of treatment regardless of the pharmacological agent selected.

Recent evidence from the OASIS-4 trial further supports the effectiveness of oral incretin-based therapy, demonstrating that oral semaglutide 25 mg achieves clinically significant weight loss and expands treatment options for patients who prefer oral over injectable anti-obesity medications (119).

An additional consideration is the difference between efficacy demonstrated in randomized controlled trials and effectiveness in routine clinical practice (117, 121). Clinical trials generally involve intensive follow-up, structured dose escalation, adherence monitoring, and standardized lifestyle counselling, whereas real-world treatment may be affected by medication cost, access, discontinuation, comorbidities, patient preferences, and variable adherence. Consequently, the magnitude of weight loss observed in clinical trials may not be fully reproduced in routine practice. Future comparative studies should therefore incorporate real-world evidence and longer follow-up to determine the durability, safety, and cost-effectiveness of newer therapies (121).

## 7 Future perspectives

The management of obesity is rapidly evolving from simple calorie restriction toward therapies targeting the biological mechanisms that regulate appetite, satiety, energy expenditure, and metabolism. Advances in molecular pharmacology, precision medicine, and digital health are expected to further improve long-term treatment outcomes (122–125).

### 7.1 Multi-receptor agonists

Emerging anti-obesity therapies are designed to target multiple hormonal pathways simultaneously, aiming to improve weight reduction and metabolic outcomes. Agents targeting GLP-1, GIP, glucagon, and amylin receptors have remarkable efficacy in reducing body weight while improving cardiometabolic health (81, 122). These multi-receptor approaches have demonstrated substantial efficacy in clinical development and may expand therapeutic options for severe obesity, although their long-term comparative effectiveness, safety, and cost-effectiveness remain to be established (81, 122).

Despite their promising efficacy, multi-receptor agonists require careful evaluation beyond short-term weight reduction. Their long-term safety, cardiovascular and metabolic benefits, effects on body composition, treatment persistence, and comparative effectiveness against existing therapies remain important areas of investigation (81, 122). Furthermore, increasingly complex pharmacological strategies may increase treatment cost and potentially complicate treatment selection. Future studies should therefore determine whether greater pharmacological efficacy translates into meaningful improvements in long-term morbidity, mortality, quality of life, and health-system outcomes (122).

### 7.2 Oral anti-obesity therapies

Although injectable incretin-based therapies have shown exceptional efficacy, the need for regular injections may reduce long-term adherence. The development of oral non-peptide GLP-1 receptor agonists such as orforglipron may provide a more convenient alternative to injectable therapies (122). Recent phase III evidence from the ATTAIN-1 trial demonstrated clinically meaningful weight reduction with once-daily oral orforglipron in adults with obesity without diabetes. At 72 weeks, mean body-weight reduction was 7.5%, 8.4%, and 11.2% with orforglipron doses of 6, 12, and 36 mg, respectively, compared with 2.1% with placebo. However, long-term treatment persistence, comparative effectiveness, and longer-term safety require continued evaluation (122). The development of oral incretin therapies may address an important limitation of injectable treatment, particularly for individuals who prefer oral administration. Nevertheless, convenience alone does not establish clinical superiority. The long-term adherence, gastrointestinal tolerability, efficacy relative to injectable agents, pharmacokinetic considerations, and affordability of oral therapies will determine their eventual role in obesity management (122, 123).

### 7.3 Precision medicine

Precision medicine represents a potentially important future direction because individuals with obesity differ substantially in biological phenotype, appetite regulation, metabolic risk, comorbidities, and response to pharmacotherapy. Integration of genetic, metabolic, hormonal, behavioural, and clinical characteristics may eventually allow prediction of therapeutic response and adverse effects (123). However, most proposed biomarkers have not yet been sufficiently validated for routine clinical decision-making. Future research should therefore focus on prospectively validating predictive biomarkers and determining whether biomarker-guided treatment improves clinical outcomes compared with conventional treatment selection (123).

### 7.4 Digital health and artificial intelligence

Digital health and artificial intelligence may facilitate continuous monitoring, behavioural intervention, treatment adherence, and individualized lifestyle support (124, 125). However, their clinical utility remains dependent on data quality, patient engagement, interoperability, algorithmic validity, privacy, and equitable access to digital technologies. AI-based approaches should therefore be evaluated not only for predictive accuracy but also for clinical effectiveness, safety, cost-effectiveness, and potential disparities in access (124, 125).

### 7.5 Emerging therapeutic targets

Several novel therapeutic targets are currently under investigation, including:

- Fibroblast growth factor-21 (FGF-21)
- Growth differentiation factor-15 (GDF-15)
- Melanocortin-4 receptor (MC4R)
- Brown adipose tissue activation
- Gut microbiota modulation
- Gene- and RNA-based therapies

These strategies may further expand future therapeutic options for obesity (10). Although these emerging targets provide promising opportunities for future drug development, translation from mechanistic or preclinical findings to clinically meaningful weight reduction remains challenging. Target validation in humans, appropriate patient selection, long-term safety, durability of response, and demonstration of clinically relevant cardiometabolic benefits will be necessary before these approaches can be incorporated into routine obesity care (10).

### 7.6 Challenges and future directions

Despite significant advances, several important challenges remain:

- Substantial weight regains following discontinuation of pharmacotherapy (98)
- High cost and limited accessibility of newer anti-obesity medications (15, 15)
- Inadequate insurance coverage and persistent social stigma associated with obesity (15, 20).
- Limited long-term safety data for emerging therapies (81, 122).
- Variability in individual treatment response (123).

These challenges indicate that the next phase of obesity pharmacotherapy should not be defined solely by the development of drugs capable of producing greater weight loss. An effective long-term strategy must also address treatment persistence, weight regain after discontinuation, adverse effects, affordability, accessibility, and individual variability in response (15, 15, 81, 98, 123). Because obesity is a chronic disease, discontinuation of effective pharmacotherapy may be followed by substantial weight regain, emphasizing the need to develop sustainable maintenance strategies rather than short-term weight-loss interventions (98). In addition, the high cost and unequal availability of newer therapies may widen existing health disparities if access is restricted to selected populations (15, 15).

Future clinical research should prioritize long-term head-to-head comparisons, real-world effectiveness, cardiovascular and other clinical outcomes, cost-effectiveness, body-composition changes, quality of life, and strategies for maintaining weight loss after treatment initiation or discontinuation (15, 15, 81, 98). Integration of pharmacotherapy with dietary intervention, physical activity, behavioural therapy, digital health, and appropriate metabolic surgery will likely remain essential for comprehensive obesity management (15, 124, 125).

Future obesity management is likely to move toward more individualized, phenotype- and mechanism-based treatment through integration of genetic, metabolic, behavioural, and clinical biomarkers with advanced pharmacotherapy (123). Oral small-molecule incretin therapies, multi-receptor agonists, FGF21- and GDF15-based approaches, adipose-tissue targeting, and microbiome-directed interventions may expand therapeutic options; however, their long-term efficacy, safety, affordability, and real-world effectiveness require further evaluation (10, 81, 122). Sustainable obesity care will also require strategies to address weight regain after treatment discontinuation, equitable access to effective therapies, and integration of pharmacotherapy with behavioural and digital health interventions (15, 98, 124, 125).

### 7.7 Public health implications and population-level strategies

Obesity represents a major and growing public health challenge that cannot be addressed through individual-level treatment alone. Although pharmacotherapy can produce clinically meaningful weight reduction in appropriately selected individuals, the development and persistence of obesity are influenced by environmental, socioeconomic, behavioural, and structural determinants. Therefore, effective obesity control requires an integrated approach combining individual clinical management with population-level prevention and interventions targeting the environments in which people live, work, and obtain food (126, 127).

Population-level prevention should focus on creating environments that facilitate healthy dietary and physical activity behaviours. Strategies include improving access to healthier foods, reducing exposure to energy-dense and nutrient-poor foods, promoting healthy dietary patterns, strengthening nutrition education, and implementing evidence-based interventions within schools, workplaces, and communities. However, interventions involving commercial actors and food industries should be carefully evaluated because evidence regarding their effectiveness in improving food environments and population health remains mixed. Strong implementation, monitoring, transparency, and accountability are therefore important when developing food-environment policies (126, 127).

The built environment is also an important determinant of obesity risk because neighbourhood characteristics can influence opportunities for physical activity. Evidence indicates that physical activity partially mediates the relationship between the built environment and obesity, with more walkable environments potentially supporting greater physical activity and healthier population outcomes. Accordingly, population-level strategies should include development of walkable neighbourhoods, safe pedestrian and cycling infrastructure, accessible recreational spaces, active transportation, and community-based opportunities for physical activity. Nevertheless, the effects of environmental interventions are context-dependent, and further longitudinal research is required to determine which specific environmental modifications provide the greatest population-level benefit (126).

Early-life prevention represents another important component of long-term obesity control. Prevention strategies should begin during pregnancy and continue throughout infancy, childhood, and adolescence, with emphasis on healthy dietary practices, appropriate physical activity, reduction of sedentary behaviour, adequate sleep, and supportive family and school environments. Schools can provide an important setting for implementing nutrition and physical activity interventions because they provide access to large numbers of children and adolescents during critical periods of growth and behavioural development. A life-course approach may therefore complement individual treatment by reducing the development of obesity and its metabolic consequences at an earlier stage (127).

The increasing availability of highly effective anti-obesity medications also raises important issues regarding health equity. Although newer pharmacotherapies can produce substantially greater weight reduction than many earlier agents, access to obesity treatment is not distributed equally across populations. Recent evidence indicates disparities in obesity-treatment access and utilization across socioeconomic status, race and ethnicity, rurality, sex and gender, and disability status. These findings suggest that improvements in pharmacological efficacy alone may not translate into equitable population-level benefits unless barriers at individual, healthcare-system, and policy levels are addressed (128).

Affordability and availability of anti-obesity medications represent additional challenges. High medication costs, inadequate insurance coverage, supply shortages, and difficulties with prior authorization may restrict access to pharmacological treatment. Pharmacists and other healthcare professionals may have an important role in improving medication access, assisting with insurance and affordability issues, supporting adherence, and providing ongoing medication and lifestyle counselling (129). Consequently, future implementation of obesity pharmacotherapy should consider not only efficacy and safety but also affordability, accessibility, treatment persistence, and the capacity of healthcare systems to provide long-term follow-up.

Economic considerations are particularly relevant because obesity pharmacotherapy is generally intended for chronic use. A recent systematic review of economic evaluations found substantial variation in the cost-effectiveness of anti-obesity medications according to drug, comparator, healthcare setting, and economic assumptions. Although highly effective newer therapies may provide substantial clinical benefits, their economic value may differ across healthcare systems and resource settings. Cost-effectiveness analyses should therefore be incorporated into policy and reimbursement decisions, particularly where healthcare resources are limited (130).

Finally, public-health strategies should recognize obesity as a chronic disease rather than simply a consequence of individual behavioural choices. Pharmacotherapy should complement, rather than replace, dietary intervention, physical activity, behavioural support, prevention programmes, and appropriate metabolic surgery. Sustainable progress will require coordinated action across healthcare systems, schools, workplaces, communities, food environments, and policy sectors. The future success of obesity management should therefore be evaluated not only by the magnitude of weight loss achieved by individual therapies but also by treatment durability, reduction in obesity-related complications, affordability, accessibility, health equity, quality of life, and broader population-level health impact (127, 128, 130, 131).

### 7.8 Unresolved questions and research priorities

Despite major advances in obesity pharmacotherapy, several important questions remain unresolved. First, the optimal duration of pharmacological treatment and the most effective strategies for maintaining weight loss after treatment discontinuation remain uncertain (15, 98). Second, comparative evidence between newer agents is still limited, particularly for long-term clinical outcomes rather than weight reduction alone (81, 122). Third, the long-term safety of recently developed multi-receptor agonists and other investigational therapies requires continued surveillance (10, 81, 122).

Further uncertainty exists regarding the extent to which trial-based efficacy can be reproduced in routine clinical practice, where treatment access, affordability, adherence, comorbidities, and patient preferences may differ substantially from those observed in randomized controlled trials (15, 15). In addition, the identification of reliable biomarkers for predicting treatment response remains an important unmet need (123). Future research should therefore integrate randomized clinical trials, long-term extension studies, real-world evidence, pharmacoeconomic analyses, and precision-medicine approaches (15, 15, 81, 123).

Finally, pharmacological advances should be considered within the broader public-health context of obesity. Medication alone cannot address the environmental, socioeconomic, behavioural, and structural determinants that contribute to obesity. Sustainable progress will require integration of effective pharmacotherapy with prevention strategies, healthy food environments, physical activity promotion, behavioural interventions, and equitable access to evidence-based healthcare (15, 15, 20).

## 8 Conclusion

Obesity is a chronic, multifactorial, and relapsing disease that requires comprehensive, long-term management. Although lifestyle modification remains the cornerstone of therapy, pharmacological treatment has become an essential component of care for patients who do not achieve adequate weight reduction through behavioural interventions alone.

Obesity pharmacotherapy has undergone substantial transformation, with the development of GLP-1 receptor agonists, dual and multi-receptor agonists, and other emerging therapies providing substantially greater weight reduction than many earlier pharmacological approaches. These advances have also expanded the therapeutic potential of obesity treatment beyond weight reduction to include improvements in glycaemic control and other cardiometabolic outcomes. However, the magnitude of weight loss reported in clinical trials should be interpreted in the context of differences in study populations, treatment duration, adherence, background lifestyle interventions, and outcome definitions, and direct comparisons should preferentially rely on head-to-head evidence.

Despite these advances, important challenges remain. Obesity is a chronic and relapsing disease, and weight regain following treatment discontinuation highlights the need for long-term maintenance strategies. Gastrointestinal and other adverse effects, treatment persistence, high medication costs, limited accessibility, variable treatment response, and differences between clinical-trial efficacy and real-world effectiveness continue to influence therapeutic outcomes. Furthermore, long-term safety and comparative clinical outcomes remain incompletely characterized for several recently developed and investigational therapies.

Future obesity management will therefore require a shift from selecting therapies solely according to the greatest percentage of weight loss toward individualized treatment based on efficacy, safety, comorbidities, treatment preferences, affordability, accessibility, and expected long-term benefit. Precision medicine, multi-receptor and combination therapies, oral incretin approaches, digital health, and biomarker-guided treatment may further improve therapeutic personalization. Nevertheless, these approaches require rigorous validation through long-term comparative studies and real-world evidence.

Ultimately, pharmacotherapy should be viewed as one component of comprehensive obesity care rather than a substitute for lifestyle and public-health interventions. Sustainable improvement in obesity outcomes will require integration of effective pharmacological treatment with dietary modification, physical activity, behavioural support, appropriate metabolic surgery, prevention strategies, and equitable access to healthcare. Addressing these clinical, economic, and public-health challenges will be essential to translate recent pharmacological advances into durable and meaningful improvements in the health of individuals and populations with obesity.

## 9 Limitation of the study

This review primarily summarizes published evidence and may not include unpublished trial data. Several emerging agents remain under investigation, and long-term safety and real-world effectiveness are still evolving. Future updates will be required as additional phase III studies and regulatory approvals become available.
